# Oxygen Biosensors and Control in 3D Physiomimetic Experimental Models

**DOI:** 10.3390/antiox10081165

**Published:** 2021-07-22

**Authors:** Jorge Otero, Anna Ulldemolins, Ramon Farré, Isaac Almendros

**Affiliations:** 1 Unitat de Biofísica i Bioenginyeria, Facultat de Medicina i Ciències de la Salut, Universitat de Barcelona, 08036 Barcelona, Spain; jorge.otero@ub.edu (J.O.); anna.ulldemolins@ub.edu (A.U.); rfarre@ub.edu (R.F.); 2 Centro de Investigación Biomédica en Red, Enfermedades Repiratorias, 28029 Madrid, Spain; 3 Institut de Nanociència i Nanotecnologia UB, 08028 Barcelona, Spain; 4 Institut d’Investigacions Biomèdiques Agustí Pi i Sunyer, 08036 Barcelona, Spain

**Keywords:** oxygen biosensors, hypoxia, oxidative stress, physiomimetic experimental models, bioreactors

## Abstract

Traditional cell culture is experiencing a revolution moving toward physiomimetic approaches aiming to reproduce healthy and pathological cell environments as realistically as possible. There is increasing evidence demonstrating that biophysical and biochemical factors determine cell behavior, in some cases considerably. Alongside the explosion of these novel experimental approaches, different bioengineering techniques have been developed and improved. Increased affordability and popularization of 3D bioprinting, fabrication of custom-made lab-on-a chip, development of organoids and the availability of versatile hydrogels are factors facilitating the design of tissue-specific physiomimetic in vitro models. However, lower oxygen diffusion in 3D culture is still a critical limitation in most of these studies, requiring further efforts in the field of physiology and tissue engineering and regenerative medicine. During recent years, novel advanced 3D devices are introducing integrated biosensors capable of monitoring oxygen consumption, pH and cell metabolism. These biosensors seem to be a promising solution to better control the oxygen delivery to cells and to reproduce some disease conditions involving hypoxia. This review discusses the current advances on oxygen biosensors and control in 3D physiomimetic experimental models.

## 1. Physiomimetic In Vitro Models

The study of diseases and the development of new therapies are currently being hampered by the use of the preclinical animal models available. In addition to the ethical concerns that arise from their use in research, animal models often give a poor prediction for the responses of subsequent human clinical trials [1]. In vivo, cells within organs are three-dimensionally organized, and they have intricate physicochemical interactions with the surrounding cells and with their microenvironment. The use of animal models has the advantage that all real-life cell interactions are included, but it has the limitations inherent to being carried out in species other than human. Remarkably, the use of human cells in animals requires immunosuppression treatment.

In vitro models, on the other hand, do not generally reflect the complexity of the cellular microenvironment found in vivo: two-dimensional plastic cultures, which historically have been the first choice for in vitro assays, are known to present a nonphysiological microenvironment for the cells, potentially leading to abnormal cellular function and thus limiting the applicability of the obtained results [2]. Moreover, in vitro tests usually are carried out under nonphysiological conditions and without the interactions with other cell types experienced in the organism. Thus, there is a current need for the development of physiomimetic in vitro models that permit the analysis of intercellular and tissue interactions in a more relevant organ context [3].

## 2. Extracellular Matrix Scaffolds for Experimental Models

The in vivo native microenvironment of the cells is the extracellular matrix (ECM). ECM is a complex bioactive scaffold composed by cell-generated proteins and polysaccharides arranged in a specific manner that gives tissues and organs their particular properties and mechanical structure [4]. The ECM also dictates cell fate (e.g., migration and differentiation). In the last decades, a high number of natural and synthetic biomaterials have been studied to develop ECM-mimicking scaffolds for cell culture [5]. However, the complexity of the native ECM of every tissue is very high, with thousands of different components structured in a specific way, being almost impossible to engineer an ECM-mimicking biomaterial starting from single components. Thus, the most advanced strategy to date is using the ECM of animal or human tissues as the basis for the development of the biomimetic scaffolds to be used for cell culture in a more physiomimetic environment. There are well-established protocols for the obtention of acellular ECM from almost every relevant organ or tissue in the body [6]. These decellularization protocols are being continuously improved to achieve their goal: efficiently removing the cellular material and ideally preserving the components and structure of the native ECM [7]. Organ/tissue scaffolds obtained by decellularization can be directly used for cell culture [8], or they can be engineered to produce hydrogels [9], which are hydrophilic polymeric networks holding vast quantities of water. Although the structure of the native tissue is lost during the process, hydrogels present the advantage of forming homogeneous scaffolds for 3D culture [10]. Moreover, with the evolution of 3D bioprinting technologies [11], complex 3D cultures based on hydrogels can be developed in an automated way, thus allowing better reproducibility in in vitro experiments.

In spite of efforts focused on mimicking the native ECM, especially with biomaterials developed from decellularized tissues and organs, using the current technologies it is not possible to build a functional complex organ which can be used for transplantation [12]. Nevertheless, engineered organs and tissues are highly valuable in drug testing [13] and disease modeling [14]. Besides, by using cell cultures in ECM-mimicking scaffolds, the mechanisms governing the interactions between cells and their microenvironment can be studied in depth [15]. Specifically, it is currently well known that physical ECM properties, such as stiffness [16] or gas diffusivity [17], influence the cell response. Accordingly, the biophysical signals need to be considered in the same way as chemical stimuli and be well controlled in optimal in vitro studies.

## 3. Physioxia: In Vitro Conditions Reproducing Physiological Oxygen Values

Oxygen is an essential molecule for correct cell function and survival. Cellular respiration is the main mechanism by which the cells obtain the energy needed to maintain their vital activities. However, oxygen demand strongly depends on cell type and its metabolic state. In humans, oxygen is distributed to the cells in different organs by a well-organized vascular tree. This distribution, which is exquisitely regulated by the organism, depends on the metabolic requirements of each organ in each specific situation. The oxygen partial pressure (PtO_2_), a key variable in the physiology of a tissue, results from the balance between oxygen delivery and its demand [18]. Consequently, every healthy organ and tissue is characterized by a specific PtO_2_ value which defines their physioxia status [19]. PtO_2_ values can range from ~10/30 mmH_2_O encountered in less active and vascularized tissues such as the skin to ~100 mmH_2_O in lung capillaries [19]. In conventional cell cultures, the amount of oxygen delivered to the cells is regulated by the incubator. The oxygen and nutrients are dissolved in the culture media and the gases can reach the cells by diffusion. Despite knowing the different physioxia statuses for a variety of the cells in the human body, most studies are still applying ~20% O_2_ (~152 mmHg) in cell culture [18]. Thus, it is important to realize that these experiments in so-called “normoxia” might be dangerously misleading because from the viewpoint of cell culture, the experimental condition approaches actual hyperoxia.

## 4. Hypoxia and Oxidative Stress

Free electrons generated by oxidative metabolism are accepted by the molecules of oxygen producing reactive oxygen species (ROS) such as superoxide O_2_^●−^, hydroxyl radical (HO^●^) and hydrogen peroxide H_2_O_2_. Mitochondrial ROS formation can be also enhanced by the uncoupling of electron transport [20]. Hypoxia-inducible factors (HIFs) are heterodimeric transcription factors, consisting of an oxygen-regulated α subunit (HIF-1α) and a constitutively expressed β subunit (HIF-1β) that exert pivotal roles in inducing cellular responses to hypoxia [21]. When oxygen is present, the α subunit is hydroxylated by prolyl hydroxylases (PHDs) and targeted for degradation via the ubiquitin-proteasome pathway. However, PHD activity is inhibited under normoxia and the HIF-1α can translocate into the nucleus and dimerize with the β subunit [22] activating transcription of relevant genes [23]. The stabilization of HIFs and their activation during hypoxia help to optimize ATP production and are associated with ROS formation [24]. In this context, HIF activation can reduce ROS formation by inhibiting the tricarboxylic acid cycle in the mitochondria or increase it via NADPH oxidase [24]. In physioxia, small amounts of ROS are produced, acting as signaling molecules which can be easily eliminated. However, alterations in oxygen concentrations can affect ROS formation causing oxidative stress [25]. In fact, it has been described that hypoxia and reoxygenation-induced ROS can contribute to HIF stabilization. Accumulation of ROS can interact with other macromolecules which can alter their properties, promote protein dysfunction and result in cell death [24].

Hypoxia is a hallmark of several pathological conditions. Severe chronic hypoxia and/or intermittent hypoxia have been commonly associated with inflammatory processes in a variety of diseases. For instance, HIFs have key roles in solid tumors, metabolic and cardiovascular diseases including ischemia, obesity, type-2 diabetes mellitus and nonalcoholic fatty liver disease, and most importantly, hypoxia is one of the main characteristics of respiratory diseases where HIFs have a pivotal role [26,27,28,29]. Even though HIFs induction was initially described in the context of cellular responses to reduced oxygen tension, its activation is not restricted to hypoxia [30,31,32]. HIFs are involved in innate and adaptive immune activation. For instance, HIF-1α increases the expression of a series of proinflammatory cytokines [33,34], and the expression of cytolytic molecules including granzyme B and perforin in CD8 lymphocytes T cells [35,36].

Although most studies have mainly focused on deleterious consequences, hypoxia and HIF expression could also contribute to enhance the mechanisms repairing the injured cardiovascular system. Indeed, published data support the notion that the hypoxic stimulus could mobilize mesenchymal stromal cells (MSCs) and other progenitor cells from the bone marrow [37,38,39,40,41]. For instance, HIFs promote the expression of genes associated to angiogenesis and modulate the differentiation of stem cells onto certain cell phenotypes [42]. Thus, hypoxia is an important factor to be considered in tissue engineering and regenerative medicine.

## 5. Oxygenation in 3D Experimental Models

The development of 3D in vitro models has been one of the most important advances during these last years. Natural tissue-derived ECM gels provide biochemical and structural cues that resemble the essential characteristics of the in vivo microenvironment. Accordingly, 3D experimental models are more physiologically relevant and have provided novel information about processes such as cell differentiation, morphogenesis, migration, and invasion in different fields of study. As expected, their evident advantages as compared with conventional 2D cell culture systems have increased the interest for these 3D models in drug discovery and tissue engineering [43]. For research purposes, they are feasible alternative models, given the shortage of human donor organs [44,45]. Despite these advantages, most of the available 3D systems lack vascularization networks. In this context, the supply of oxygen and nutrients as well as the removal of CO_2_ and metabolic waste products are the main problems in nonvascularized engineered tissues [46]. In fact, the typical diffusion limit considered for most tissues is around 200 μm being the smallest autonomous unit in the absence of vascularization [46,47].

As occurs in 2D cell cultures, in the 3D experimental models oxygenation and the supply of nutrients such as glucose are based on gradient-driven passive diffusion. Despite oxygen having a higher diffusion coefficient than glucose and a parallel consumption rate, its lower solubility in culture media makes it a key limiting factor in 3D constructs [48]. Oxygen solubility in a typical culture medium is 0.2 mmol O_2_/L when atmospheric oxygen is used, half to the solubility reported in pure water [49]. Diffusion of O_2_ could be increased by augmenting oxygen gas content, however this would induce the presence of free radicals in the culture medium, which are cytotoxic for cells [50]. Thus, the cell culture medium must be continuously in circulation and re-oxygenated by using an in-line gas-exchanger [51,52]. The experimental setup employed should reproduce the oxygen tension and nutrient supply characteristic for each organ/tissue, or the hypoxic environment and nutrient limitation that occurs in some situations such as in solid tumors. Therefore, there are different 3D culture experimental approaches attempting the recreation of different organs and tissues to understand their physiology and several associated pathologies.

To better understand the oxygen diffusivity in 3D gels with different compositions and density, the oxygen diffusion and consumption were studied by using a polarographic needle electrode [53] in gels which included reconstituted basement membrane (rBM), fibrin and collagen I with a thickness of 7 mm. The oxygen diffusivity in acellular gels with a density of collagen-I ≥3 mg/mL showed a reduction of 40% with respect to water, but no changes were observed in sparce ECM. That study specifically revealed a dominant effect of ECM composition over density in the oxygen consumption rate (OCR). O_2_ diffusivity was 1.01 × 10^−5^ cm^2^/s for rBM, 1.18 × 10^−5^ cm^2^/s for dense fibrin, 1.25 × 10^−5^ cm^2^/s for dense collagen-I, and 1.46 × 10^−5^ cm^2^/s for sparse collagen-I. Thus, this decrease in the diffusivity of oxygen could be attributed in part to the geometry of the scaffold [54]. In addition, oxygen tension measurements in these gels provided direct values of the maximum OCR of A549 human alveolar adenocarcinoma cells. In a similar approach [55], O_2_ diffusivity in collagen 3D constructs was measured. Collagen sheets were assembled by rolling to obtain a 3D spiral construct and sealed to measure material and scaffold perfusion. Then, a luminescent oxygen probe was inserted into the core of the construct and then oxygen in the external medium was replaced by bubbling N_2_ (O_2_ changing from 21% to 0%). The resulting O_2_ time course plot provided information about mass transport. These measurements showed that the collagen construct was equilibrated to the O_2_ tension of the external medium. The oxygen diffusion rate was 4.5 × 10^−6^ cm^2^/s in the single compressed native collagen construct. Photochemical crosslinking of the collagen scaffold per se induced a reduction of ~24% (3.4 × 10^−6^ cm^2^/s). However, increasing three-fold the collagen density produced a marked reduction in O_2_ diffusion ~62% (1.7 × 10^−6^ cm^2^/s).

**Figure 1:**
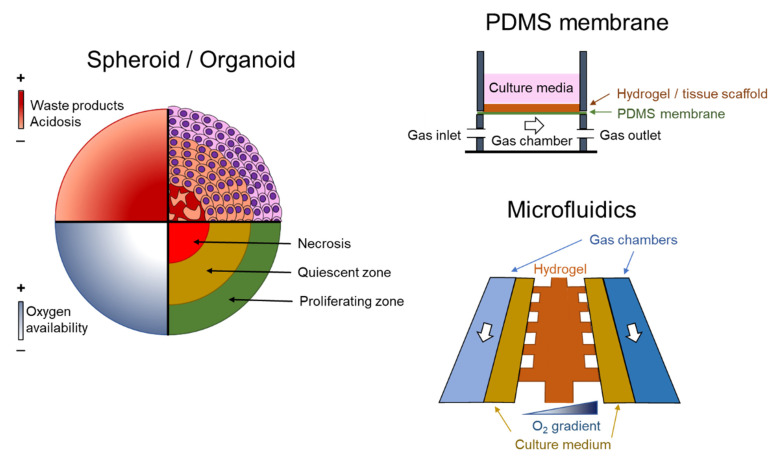
Examples of experimental models. On the left, the absence of vascularization in spheroids and organoids causes a heterogeneous distribution of oxygen, waste products and acidosis. These structures are characterized by a well-irrigated proliferating layer in the periphery, a quiescent layer and a necrotic core. On the right, two methods to create a spatiotemporal O_2_ distribution. At the top, a semipermeable polydimethylsiloxane (PDMS) membrane is able to produce fast changes in O_2_ in cell culture, and at the bottom, by using microfluidic devices.

Other options in 3D culture are the use of spheroids and organoids [56], two very similar experimental approaches. Organoids are complex clusters of organ-specific cells, usually stem cells or progenitor cells, which can self-assemble within an ECM environment. In contrast, spheroids do not require a scaffolding to form 3D cultures. As occurs in 3D substrates, both systems of culture also present a diffusion-limited supply of oxygen. As in 3D hydrogel scaffolds, the measurement of oxygen gradients in spheroids has been carried out with microelectrodes, EF5 (a 2-nitroimidazole-based molecule) and pimonidazole [57,58,59]. As expected, a diffusion-limited supply of oxygen results in a marked gradient from the periphery to the core (Figure 1). Although the oxygen polarographic microelectrode is the most used method, it has some limitations such as oxygen production/consumption, signal drift, media requirements and disruption of the structure [60]. A noninvasive approach, based on electron paramagnetic resonance (EPR) oximetry to quantify and evaluate the formation of oxygen gradients in spheroids developed from RTG-2 cells (a nonmalignant fish cell line) has been described [61]. This work provides novel insights into the size and cell seeding densities appropriate for use in non-tumor studies. EPR is widely employed to measure free radicals and oxygen availability in tissues, and it does not destroy the oxygen gradients as when using microelectrodes [61,62].

## 6. Spatiotemporal Oxygen Heterogeneity in 3D Cultures

The limited diffusion and solubility of gases in 3D constructs make it difficult to expose cells to well-defined spatial and temporal variations in oxygen tension. For instance, the tumor microenvironment is characterized by aberrant vascularization leading to intermittent hypoxia in some regions. In cancer, the behavior of tumor cells can be modulated by hypoxia, promoting tumor growth and cancer metastasis [63,64,65]. Other pathologies such as obstructive sleep apnea elicit intermittent hypoxia which has been linked to oxidative stress and inflammation in some tissues and organs [66,67,68]. Therefore, reproducing the specific spatiotemporal oxygen heterogeneity observed in any pathology associated with impaired local oxygenation is essential to better understand the role of hypoxia. To this end, interesting experimental settings allowing the application of controlled gradients of oxygen and cyclic changes of gases have been developed.

During the last decade, the number of systems allowing the control of oxygen tension in cultured cells under spatiotemporal oxygen heterogeneity has increased. For instance, a microfluidic device capable of controlling both the spatial and temporal variations in oxygen tensions consists of three parallel connected tissue chambers and an oxygen scavenger channel placed adjacent to these tissue chambers [69] (Figure 1). Cultured endothelial and fibroblasts can form a 3D vascular network in the central chamber and a spatial and temporal oxygen gradient was generated by using sodium sulfite, an oxygen scavenger, in the adjacent channel. More recently, a double-layer microfluidic device to control the oxygen tension under spatiotemporal oxygen heterogeneity mimicking the tumor microenvironment has been described [70]. To this end, a couple of parallel gas channels above the media and the 3D gel channels were included to facilitate gas exchange. This device generated uniform hypoxic conditions and linear oxygen gradients across the gel channel. The use of hydrogels combined with bioprinting and stereolithography has increased the possibilities of creating novel bioreactors capable of recreating the spatiotemporal oxygen heterogeneity observed in some physiological and pathological conditions. As an example, photopolymerizable hydrogels were employed to perform intravascular and multivascular design freedoms [71]. Monolithic transparent hydrogels with intravascular 3D fluid mixers and functional bicuspid valves were created.

## 7. Bioreactors and Oxygen Control

Bioreactors are the equipment developed to control the biophysical conditions where cells and tissues are cultured [72]. In addition to controlling the standard parameters as in classical CO_2_ incubators (such as temperature or relative humidity), bioreactors are designed to mainly control gas gradients [73], mechanical stresses (static [74] and dynamic [75]) and electrical currents [76]. The main idea is to control a high number of the physicochemical stimuli to which cells are subjected within the organs and tissues in vivo [77]. Historically, bioreactors have been developed with discrete components (sensors and actuators) by adapting the available CO_2_ incubators. For applications where the gas mixture is an important parameter, dissolved oxygen should be measured within the bioreactor.

Iodometric titration, which is considered a standard for laboratory applications, is a classical test for dissolved oxygen determination [78]. As there is the need to add reagents such as manganese sulfate or potassium iodide into the solution, the method is not applicable for measuring oxygen in the case of physiomimetic settings. Moreover, the iodometric titration method does not provide continuous oxygen monitoring, hence it is only practical for calibration procedures. The alternative for measuring oxygen concentration in cell cultures is the use of real-time monitoring electrochemical or optical biosensors [79].

**Figure 2:**
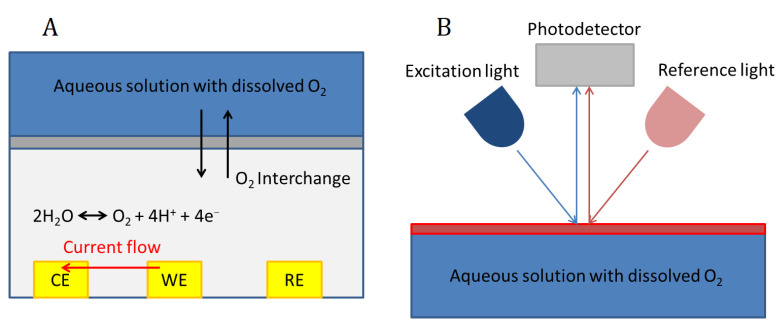
Dissolved oxygen sensors working principle. Electrochemical sensor (**A**); Optical sensor (**B**).

Electrochemical biosensors for the measurement of dissolved oxygen are based on sensing the change of the current or the potential in an electrode, or the changes in the conductance of the solution, which are dependent on oxygen concentration (Figure 2). Among them, the most widely used are current-measuring biosensors, which can use either the polarographic or the galvanic method, the first being the most widely used for bioreactors. They are mainly based on using a Clark-type electrode [80]. A Clark-type sensor consists of three electrodes (working (WE), reference (RE) and counter (CE) electrodes), an electrolyte and an oxygen-permeable membrane which are overlaid on the electrodes. Usually, a chamber is located on top of the permeable membrane to contain the solution under test. When the oxygen in the sample solution permeates through the membrane to the inner electrolyte (which can also be a solid electrolyte membrane), the reduction current of oxygen is measured at the WE.

Optical oxygen sensors are based on the optical absorption of the light by the oxygen molecules dissolved in the liquid. There are many different optical principles that can be used, but the most widely employed for biosensors is the fluorescent quenching method [81]. The principle consists of using a fluorescent substance or dye which absorbs and emits at determined wavelengths (Figure 2). The collision between oxygen molecules and the dye interferes in the excitation process, and therefore oxygen concentration can be determined by measuring fluorescence intensity or lifetime in a photodetector (Figure 2).

The selection of an electrochemical or an optical biosensor for the measurement of dissolved oxygen depends on several factors. Optical detection presents higher accuracy and lower response time, and the maintenance of the device is less frequently required. In addition, it is interesting that optical sensors do not consume oxygen in the measurement process. Although these devices seem superior in performance when compared with electrochemical biosensors, the latter are cheaper and can be more easily integrated into organ-on-a-chip devices. For the above-mentioned reasons, electrochemical biosensors, despite their inferior characteristics, are still widely used in several experimental settings as shown in Table 1.

An evolution of bioreactor technologies is the development of organ-on-a-chip models [94]. These devices try to recreate the physiological chemical and physical microenvironments of living tissues and organs [95]. Attempts trying to reproduce almost all organs in the human body by means of organ-on-a-chip have been reported nowadays by different research centers or companies worldwide [96]. These microphysiological systems are microfluidic lab-on-a-chip devices integrating sensors and actuators. The application of dynamic fluid flow for the physiological nutrition of the tissues and for the creation of microenvironmental biomolecular gradients, and the control of relevant biophysical cues (such as mechanical strains, oxygen gradients, and electric stimuli) are the major aspects that differentiate organ-on-a-chip devices from conventional cell and tissue cultures. The measurement and control of oxygen in organ-on-a-chip systems require the incorporation of integrated sensors into these devices. The generation of oxygen gradients in physiomimetic systems is challenging, considering the different diffusivity of gases in those materials of which the device is composed. Most of these devices are fabricated by using polydimethylsiloxane (PDMS) because it can be easily fabricated by soft lithography [97]. PDMS has a high permeability to gases. Although this characteristic is an important hallmark for several biological applications, the use of PDMS makes it more difficult to precisely control the oxygen concentration. To decrease its diffusion coefficient and improve oxygen control, PDMS can be coated with parylene-C [98]. As a substitute of PDMS, polymethyl methacrylate (PMMA) can be used [99]. Thus, combining low- and high-diffusive materials, a gradient of oxygen can be obtained within the same device. Oxygen gradients can be also generated by using hydrogels tuned for low-oxygen diffusivity [100] or by controlling oxygen delivery by microfluidics. For instance, when flow is very low, oxygen is mainly transported by diffusion resulting in a gas gradient between the inlet and outlet of the device [101]. Therefore, taking into consideration the different diffusivity of the materials and experimental approaches, spatiotemporal oxygen monitoring is important.

Oxygen monitoring should be done by integrating biosensors in the physiomimetic device. Electrochemical oxygen sensors can be implemented by microfabrication facilities for lab-on-a-chip applications [102], using membranes based on agar [103] or polyacrylamide [104] and Ag/AgCl as material for the electrodes. There are different methods for integrating the electrodes into the system. These sensors can be composed of simple planar electrodes [105] or they can be distributed into the device as microarrays, thereby, the profiles of oxygen can be monitored [106]. In the case of optical biosensors, optically responsive particles can be used as the dye for the measurement within organ-on-a-chip devices [107]. The first strategy for the measurement is to incorporate oxygen sensitive dyes into the device during the fabrication process and to measure their response by using an external probe [108]. Alternatively, dissolved dyes in the culture media can be perfused [109]. The position of the sensor in the device (either electrochemical or optical) is crucial. For instance, the oxygen values measured strongly depend on the distance to cells [110]. More advanced systems can include a feedback loop to control oxygen concentration and/or gradients by adjusting the N_2_-O_2_ concentrations in a gas introduced into the device by a gas blender [111].

Most research on oxygen control in organ-on-a-chip devices has been conducted in the context of developing respiratory models. Lung-on-a-chip models can not reflect the structure and function of the intricate alveolar-capillary system by using current fabrication technologies [112,113]. Available systems are based on the coculture of endothelial and epithelial cells separated by a gas-permeable membrane, and on the inclusion of a system for mechanically stretching the culture to simulate lung breathing. In this way, mechanical forces (cyclic stress produced by lung breathing and shear stress produced by blood flow in the capillaries) can be under control in in vitro experiments. Recent developments in the lung-on-a-chip field have incorporated oxygen as another parameter under control in these miniaturized bioreactors. As the mechanical actuators are usually pneumatically driven, gas concentration can be changed in the lung-on-a-chip device by modifying the mixture of gases that is introduced to the device for activating the mechanical stretch. Most of the technological developments in this field have been focused on studies related with cancer, as hypoxia has been widely associated with tumor progression and aggressiveness [114]. Some other works have focused the bioreactor design on the generation of oxygen gradients. For instance, oxygen was controlled to determine how the alveolar cell migration capacity varies when subjected to different oxygen gradients [115].

## 8. Conclusions and Perspectives

Physiomimetic experimental models are quickly evolving thanks to the advances in organ-on-a-chip and tissue engineering technologies. As the lung is the organ where gas concentration changes are of major interest, most of the efforts are being made in mimicking the pulmonary system in physiomimetic models. Lung-on-a-chip devices have been developed with the focus on mechanically stretching the cells, but recently more efforts have also been devoted to controlling the oxygen concentration within the devices [114,116]. It is also worth noting that much progress is expected from the fast evolution of 3D bioprinting technologies. Although nowadays this technology cannot reproduce a full organ such as the lung (and it is not expected to in the next few years [12], a recent report described the implementation of alveolar-like structures where oxygenation of red blood cells can be monitored in vitro [71]. Regarding other tissues and organs, O_2_ monitoring is important for tissue engineering and regenerative medicine because stem cell differentiation, cell therapy and engraftment of progenitor cells into the target tissue seem to be more efficient at specific levels of oxygen [117,118]. Although the measurement of dissolved oxygen is considered a good indicator and can be used to generate an oxygen gradient, it is not always a good estimator of cellular oxygen levels [110]. This limitation could be solved by designing novel devices using distributed integrated biosensors and by using computational models to estimate oxygen cellular levels from dissolved oxygen measurement [119]. The development of such sophisticated physiomimetic systems incorporating oxygen control will provide: (i) new opportunities to better understand human physiology, (ii) the improvement of cell therapy and regenerative medicine and (iii) an understanding of how oxygen deregulation could explain the development and progression of multiple diseases.

## Figures and Tables

**Table 1:** Available bioreactors with real-time oxygen monitoring.

Organ/Tissue	Device	Scaffold	O_2_ Sensor	Application	Reference
Lung	Chip bioreactor	None	Clark type sensor	Investigating the effects of high-rate changes in oxygen concentration in the cell culture medium	[82]
	Whole organ bioreactor	None	Optical sensor	Maintenance of constant levels of dissolved oxygen throughout culture period and its real-time quantification	[83]
Heart	Perfusion bioreactor	Decellularized murine heart	Optical sensor	Biomechanical stimulation through defined and controlled 3D stretching of the heart ventricle	[84]
	Perfusion bioreactor	Alginate scaffolds	Electrochemical sensor	Integration of electric field stimulation	[85]
Liver	Microfluidic device	None	Electrochemical sensor	Toxicology, drug-screening, personalized cancer therapy, organ-on-a-chip culture, and tissue engineering.	[86]
	Microfluidic device	None	Electro galvanic sensor	Study the role of oxygen and hypoxia-associated molecules in modelling healthy and injured liver tissues	[87]
Brain	Microfluidic device	None	Clark type sensor	Readily control oxygen depletion rates inside the biochip.	[88]
Vessel/Vascular tissue	Perfusion bioreactor	Decellularized aorta from rabbit	Optical sensor	Tissue engineering of large-scale small-diameter vascular vessels for clinical use.	[89]
Skin	Perfusion bioreactor	None	Optical sensor	Multianalyte microfluidic bioreactor to monitor pH and dissolved oxygen levels	[90]
Adipose tissue	Tissue mass culture bioreactor	Silated-hydroxypropyl-methylcellulose (Si-HPMC) hydrogel	Fluorescent sensor	Analysis of nutrient transport and gas exchange in hydrogels	[91]
Entothelial and adipose tissue	Microfluidic device	Fibrin hydrogel	Optical sensor	Control of cyclic normoxic−hypoxic cell microenvironments	[92]
Embryonic tissue	Microfluidic device	None	Optical sensor	Analyze growth and oxygen uptake kinetics in real time.	[93]
